# Composition of Bread
*On the Composition of Bread. By Douglas Maclagan, M.D., F.R.S.E. Read before the Chemical Section, British Association, September 18, 1855. Pamphlet, pp. 14. Edinburgh: 1855.


**Published:** 1856-04

**Authors:** 


### COMPOSITION OF BREAD.*

The determination of the amount of water and of available nutritive material in bread, is a subject to which but little attention has been paid in this country; and hence the value of bread as an article of diet has never been correctly ascertained. Dr. Douglas Maclagan finds that the amount of moisture in bread is less than has generally been calculated; but his results agree most closely with those of the French chemist, Payen. According to the last-named chemist, the percentage of moisture in French white bread is 36; and in the present ration bread of the French army, 35. Dr. Maclagan finds the average percentage in bakers' fine bread to be 35·75; in fine home-baked bread, 33·93; in bakers' coarse bread, 34·91. From his analyses, he calculates the average produce of a sack (280 lbs.) of flour to be ninety-three and a half quartern loaves, in the hands of the baker; and this agrees very nearly with the estimate given by bakers in Edinburgh, who hold ninety-two such loaves to be the average produce of a sack. In home-baking, the gain is so small (being only two quartern loaves per sack), that Dr. Maclagan doubts whether there is much economy in the domestic process. Unfermented bread contains more water (about 40 per cent.); and therefore, though suitable under certain circumstances, is not defensible on the score of economy.

The amount of azotised matters in bread has also been calculated by Dr. Maclagan. In bakers' fine bread, he finds 7·55 per cent.; in home-baked bread, 7·29; in coarse bread, 7·99. He observes that the report furnished by Drs. Alison and Christison to the Poor-Law Board places the quantity of azotised material too high—10·5 per cent. The average ratio of azotised to non-azotised material in bread of all kinds, taken together, is as 1 to 7·3. The amount of moisture does not appear to be, as some have maintained, in proportion to the amount of gluten; but may be in some way connected with the form in which the nitrogenous matter is present. Nor do the experiments of Dr. Maclagan confirm the idea that the amount of saline matter in bread influences the quantity of water; for example, in several analyses, the percentage of water ranging from 33·02 to 41·73, the ratio of

---

* On the Composition of Bread. By Douglas Maclagan, M.D., F.R.S.E. Read before the Chemical Section, British Association, September 18, 1855. Pamphlet, pp. 14. Edinburgh: 1855.

solids to one part of ash varied in the most irregular manner, from 24·9 to 74.

It is impossible to obtain an estimate of the average amount of salt in bread; since one baker differs from another in his practice, and from himself at different seasons, more salt being used with new than old flour. The result of Dr. Maclagan's inquiries, however, was that the salt was usually added in the proportion of 4 or 4½ lbs. to the sack of flour, or about 1·15 per cent. of the weight of bread.

In conclusion, Dr. Maclagan says that he has not been able to detect more than small and doubtful traces of alum in the bread of Edinburgh; and certainly not the "large crystals of alum", which have been stated to have been found in London bread.

